# Assessment of Drivers’ Mental Workload by Multimodal Measures during Auditory-Based Dual-Task Driving Scenarios

**DOI:** 10.3390/s24031041

**Published:** 2024-02-05

**Authors:** Jiaqi Huang, Qiliang Zhang, Tingru Zhang, Tieyan Wang, Da Tao

**Affiliations:** 1 Institute of Human Factors and Ergonomics, College of Mechatronics and Control Engineering, Shenzhen University, Shenzhen 518060, China; 2210295183@email.szu.edu.cn (J.H.);; 2 Physical Science and Technology College, Yichun University, Yichun 336000, China; 3 Xiamen Meiya Pico Information Co., Ltd., Xiamen 361008, China

**Keywords:** driver mental workload, physiological signals, behavioral performance, EEG, EOG, ECG, EDA

## Abstract

Assessing drivers’ mental workload is crucial for reducing road accidents. This study examined drivers’ mental workload in a simulated auditory-based dual-task driving scenario, with driving tasks as the main task, and auditory-based N-back tasks as the secondary task. A total of three levels of mental workload (i.e., low, medium, high) were manipulated by varying the difficulty levels of the secondary task (i.e., no presence of secondary task, 1-back, 2-back). Multimodal measures, including a set of subjective measures, physiological measures, and behavioral performance measures, were collected during the experiment. The results showed that an increase in task difficulty led to increased subjective ratings of mental workload and a decrease in task performance for the secondary N-back tasks. Significant differences were observed across the different levels of mental workload in multimodal physiological measures, such as delta waves in EEG signals, fixation distance in eye movement signals, time- and frequency-domain measures in ECG signals, and skin conductance in EDA signals. In addition, four driving performance measures related to vehicle velocity and the deviation of pedal input and vehicle position also showed sensitivity to the changes in drivers’ mental workload. The findings from this study can contribute to a comprehensive understanding of effective measures for mental workload assessment in driving scenarios and to the development of smart driving systems for the accurate recognition of drivers’ mental states.

## 1. Introduction

Road accidents pose a significant threat to public safety. According to a data report by the World Health Organization [1], road accidents have become the leading cause of death for the global population, with over 1.2 million people losing their lives due to traffic accidents each year. In China, traffic accidents were ranked as the sixth leading cause of death in 2019, accounting for over 250,000 fatalities [2]. Extensive research has been dedicated to investigating the root causes of road traffic accidents [3,4,5], among which, drivers’ mental states (such as mental workload (MWL)) have emerged as a primary factor. During the driving process, the excessive MWL imposed on drivers due to the abundance of information to be processed can lead to adverse conditions, such as increased driving risks and the occurrence of consequential traffic accidents. Therefore, the monitoring and assessment of drivers’ mental states could be especially important during the driving process, which could help develop effective interventions for early warnings of the occurrence of MWL-induced road accidents.

Driving is a complex cognitive task that requires drivers to obtain information and make decisions through visual and auditory sources [6]. With the development of automobile technology, it appears to be a normal configuration for automobiles to be equipped with varied in-vehicle information systems (IVIS), such as advanced driver assistance systems, in-vehicle information and entertainment systems, and smart driving systems [7,8,9,10]. Nowadays, while an increasing number of IVIS have been integrated with smart devices and sensors to deliver entertainment and information services through audio interfaces and allow for voice commands, their introduction is also likely to increase MWL on drivers as well, potentially resulting in detrimental effects on driving performance [11]. While interacting with an IVIS, drivers rely heavily on visual and auditory channels to receive entertainment and information services [7]. According to the Multiple Resource Theory [12], when the voice and visual information provided by IVIS requires drivers to process and make judgments, it may compete with their mental resources and cognitive ability to respond appropriately to the driving task. This competition is likely to increase drivers’ mental workload, potentially adversely impacting their driving performance. If the requirement for information processing (either from the main driving task or from non-driving related tasks by interacting with an IVIS, or both) exceeds drivers’ information processing capacity, it would cause overloaded mental states for them, which is likely to result in risky driving behaviors and even traffic accidents. Therefore, assessing MWL experienced by drivers when they simultaneously process voice and visual information in automobiles still represents an urgent need in the prevention of driving risk and road accidents [13,14,15].

MWL is widely considered as a multidimensional concept, and the current consensus is that it reflects the conflicting relationship between an individual’s information-processing ability and the capacity required to process information [16]. For a better understanding of MWL, it can be compared with physical workload [17]. Physical workload pertains to the strain exerted on an individual’s body during a task, while MWL emphasizes the subjective experience of an individual’s perceived workload for a given task. MWL could be influenced by various factors, such as task quantity, time pressure, environment, and individual experience. For example, novice drivers would perceive MWL differently compared to experienced drivers [18]. Therefore, MWL should be measured based on the identification of an individual’s personal MWL levels in particular environments.

There are three typical measuring techniques for assessing drivers’ MWL, including subjective measures, physiological measures, and task performance measures [17,19]. Subjective measures involve using scales to elicit subjective MWL ratings from drivers during the driving process, such as the NASA task load index (NASA-TLX) [20] and the Subjective Workload Assessment Technique (SWAT) [21]. Physiological measures, including electroencephalogram (EEG) signals [22], eye movement signals [23], electrocardiogram (ECG) signals [24], and skin conductance signals [25], can be employed to assess MWL of drivers while driving, as the change in MWL would also lead to changes for corresponding physiological activities in human body. Another common technique for MWL assessment is to measure drivers’ task performance, also known as the primary-secondary task measurement method [26]. This technique typically involves evaluating task performance from the primary driving task and secondary tasks that are usually used to induce various levels of MWL. Measures of drivers’ performance in the primary driving task, such as vehicle speed and distance from the lane, are used to evaluate drivers’ MWL during the driving process, while the performance of secondary tasks (e.g., reaction time, error rate) is generally used to determine the appropriateness of MWL task settings [15]. Overall, while subjective measures are intuitive for MWL assessment and easy to implement, these measures are likely to be subject to bias. In contrast, physiological and task performance measures, though indirectly to reflect MWL, can be recorded simultaneously during driving process and can avoid drivers’ conscious manipulation.

Previous studies have utilized the abovementioned measures to assess MWL of drivers and establish some relationships between the measures and MWL. For instance, modifying task difficulties during the driving process yields distinct subjective evaluations. As task difficulty increases, drivers’ subjective scores on MWL scale show noticeable differences [27,28]. Borghini et al.’s research revealed that the power of EEG signals (such as θ, δ, and α) is highly sensitive to different MWL states during the driving process. They also discovered that under high MWL conditions, the frontal lobe exhibits increased EEG power in the θ band, while the parietal lobe shows decreased EEG power in the α band [29,30]. In simulated driving environments, gaze fixation duration decreases with an increase in task load [31]. Heart rate and heart rate variability are also commonly used and are effective ECG measures for assessing drivers’ MWL [32]. Skin conductance level (SCL) and skin conductance response (SCR) are frequently used in electrodermal activity measurements to assess MWL. Belyusar et al. found a positive correlation between SCL, SCR, and MWL [33]. Driving performance metrics, such as vehicle speed and lateral position, were correlated with MWL. When MWL is high, vehicle speed and lateral position can change significantly. Therefore, these measures can be used to assess the workload state of drivers [34].

Despite the existing literature on the identification of effective measures for assessing MWL, several points should be addressed before existing evidence could be applied to a specific driving context. First, there remains an inconsistency in the effectiveness of measures used across different studies and driving scenarios. While some measures work well for assessing MWL in certain study scenarios, they may prove invalid in other scenarios [15,35]. Indeed, recent review studies have shown that there are no such universally effective measures that could work well for all scenarios to discriminate MWL [17,19]. This lack of universally effective measures arises from the fact that human responses to MWL, whether physiological, psychological, or behavioral, are highly dependent on task scenarios and can be influenced by task characteristics and individual differences [17,19]. However, the widely encountered auditory-based dual-task driving scenarios have not been well examined. Furthermore, most of previous studies examined MWL with only a limited number of measures. For instance, Almogbel et al. employed EEG measures only [22], and Heine et al. applied ECG measures only [36]. This makes it challenging to directly compare the effectiveness of different measures within the same scenarios. Consequently, it becomes crucial to combine multiple types of measures to form a comprehensive evaluation of MWL instead of relying solely on a few limited measures. Therefore, it is important to understand how MWL could be detected, based on typical manipulations of driving scenarios, and how a set of physiological, psychological, and behavioral measures would change by different levels of drivers’ MWL to allow for comparison within the same study scenario.

To address the limitations mentioned above, this study aims to provide a comprehensive assessment of drivers’ MWL with multimodal measures during a typical manipulation of driving scenario. A representative auditory-based dual-task driving scenario was created to simulate real-world driving conditions, where the widely used N-back task [37] was manipulated to simulate the auditory requirement from voice interaction with an IVIS while driving and to induce three distinct levels of MWL by varying task difficulty levels. Thus, the innovation of this study lies in its multimodal approach for a MWL assessment in an auditory-based dual-task driving scenario. This helps identify which measures are effective in MWL assessment, and which measures are insensitive to changes in MWL in such a scenario. This also allows for a direct comparison of the effectiveness of different measures and provides a more accurate understanding of how MWL affects drivers’ physiological, psychological, and behavioral responses. Such knowledge would improve the accurate assessment of MWL and can serve human state monitoring in future development of smart driving systems and autonomous vehicles.

The remaining structure of this article is as follows: Section 2 provides a detailed description of the methods used in this study, including the construction of the driving simulation scenario, experimental design, experimental procedures, and data analysis methods. Section 3 presents the results of the data analysis. Finally, we offer a discussion and conclusion of the research conducted in this study.

## 2. Materials and Methods

### 2.1. Participants

Considering previous research on sample size determination [34,38], we utilized the G*Power 3.1 software to calculate sample size [39]. A minimally required sample size of 20 was determined to detect a medium effect size of 0.3, with statistical power of 80% and significance level of 5%, based on repeated measures analysis of variance (ANOVA). Finally, we recruited 24 males (mean age = 24.5, SD = 2.3) to join in the experiment (We only included males as they were more easily able to wear an EEG cap and ECG devices for better acquisition of signals). All participants possessed a valid driver’s license with at least one year’s driving experience (mean driving experience = 2.6 years, SD = 1.3). None of the participants reported any prior history of neurological disorders, heart disease, or other medical contraindications. This study obtained approval from the Institutional Review Board of Shenzhen University, and all participants provided consent before their participation into the study.

### 2.2. Experimental Design and Tasks

**Figure 1:**
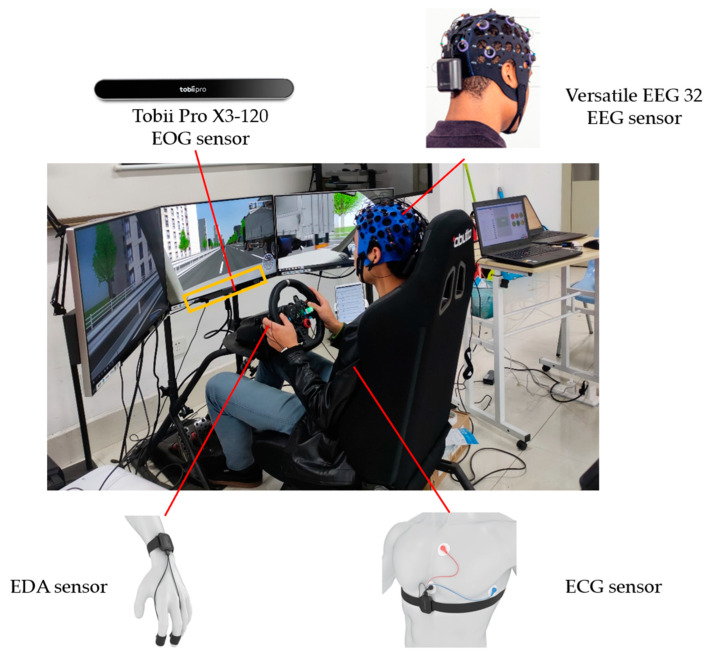
Experimental scenario and equipment for physiological signal acquisition.

We adopted an auditory-based dual-task driving scenario to simulate scenarios of verbal interaction between drivers and IVIS during the driving process, with driving tasks as the main task, and auditory-based N-back tasks as the secondary task (Figure 1). A one-factor within-subjects design was implemented in this experiment. Task difficulty served as the independent variable, including low, medium, and high levels, which was manipulated by the N-back task. The N-back task is commonly adopted to induce MWL and has been widely used in previous studies on drivers’ MWL [40,41,42]. Specifically, the low-difficulty task solely involved the primary driving task and had no presence of N-back, the medium-difficulty task encompassed both the primary driving task and a 1-back task, and the high-difficulty task included the primary driving task and a 2-back task.

The primary driving task aimed to simulate a typical driving environment in urban scenarios at a speed of approximately 80 km/h. The N-Back task was conducted in synchrony with the primary driving task through voice broadcasting of random numbers. Participants were required to respond to the N-Back tasks by pressing a button installed on the steering wheel as quickly and accurately as possible. In particular, in the 1-back task, participants were required to judge whether the current number they heard matched the one that immediately preceded it, while in the 2-back task, participants were required to judge whether the current number matched the one that preceded it by two items.

### 2.3. Apparatus and Procedures

The driving simulator utilized in this study consisted of a high-fidelity driving simulator with three monitors for the visual presentation of the driving scenario, a Logitech feedback steering wheel pedal set control system for motion control, an external speaker for audio information presentation, and a computer for behavioral data collection. FORUM8 UC-win/Road scene design 14.3 software was used to develop and present the driving scenario.

Physiological signals were synchronously recorded in real time using the ErgoLab 3.0 Platform (Kingfar, Beijing, China) that was integrated with multiple wearable devices to collect data for varied physiological signals. In particular, EEG signals were captured by a BitBrain 32-channel EEG device (Bitbrain, Zaragoza, Spain) and a Tobii Pro X3-120 eye tracker (Tobii, Stockholm, Sweden) was used to capture electrooculogram (EOG) signals, while ECG and electrodermal activity (EDA) signals were acquired through Kingfar physiological acquisition sensors (Kingfar, Beijing, China). Figure 1 illustrates the simulated driving scenario and the devices employed for physiological signal collection.

Before the experiment, participants were instructed to complete an informed consent form, and then to wear the sensors. Participants proceeded to the driving simulation platform for sufficient practice, which was aimed at familiarizing themselves with the driving simulator and experimental tasks. Then, they conducted the main test, where they were randomly assigned to one of the three driving tasks with different difficulty levels, each lasting 60 min. After the completion of each of the three driving tasks, participants were administered the NASA-TLX scale for subjective MWL assessment. To mitigate the impact of driving fatigue on the experiment, participants were required to complete the three types of driving tasks on three different days, respectively, with the order of task difficulty present in a counterbalanced Latin Square design.

### 2.4. Measures

#### 2.4.1. Subjective Ratings

The subjective evaluation of MWL was obtained through the NASA-TLX questionnaire with a rating scale ranging from 0 to 100. It encompasses six dimensions, mental demand, physical demand, temporal demand, task performance, effort exerted, and level of frustration, and has been widely used to assess MWL in varied human–computer interaction contexts [15,16]. Data on rating scores for the six dimensions were collected under varying levels of task difficulty.

#### 2.4.2. Physiological Measures

The EEG signals were preprocessed using the EEGLAB toolbox [43] and the signals of EOG, ECG, and EDA were extracted using ErgoLab 3.0. The typical processing flow for the EEG signals involved several steps. Firstly, the Kurtosis function was utilized to automatically detect and interpolate bad channels using the spherical interpolation method. Subsequently, the reference electrode on the scalp surface was selected as the average electrode for referencing. To achieve bandpass filtering (1 Hz–30 Hz), the FIR plugin was implemented. Following this, the ADJUST plugin was employed for the manual removal of artifacts such as eye and muscle activity through independent component analysis. The preprocessed brain signals were then subjected to a short-time Fourier transform (STFT) for time-frequency analysis and feature extraction. Ultimately, the study focused on extracting the average power spectra densities (PSDs) of four brain waves (δ: 1–4 Hz, θ: 4–8 Hz, α: 8–14 Hz, β: 14–30 Hz) from the EEG signals.

Regarding the processing of EOG signals, the maximum inter-blink interval was set at 75 ms. Missing data was linearly interpolated, and denoising was performed using a sliding median filter. The angular velocity of fixations was computed with a window length of 20 ms and a threshold of 30°/s. After classification, a maximum time threshold of 75 ms and a maximum angular threshold of 0.5° were set between fixations. Fixations not meeting these criteria were merged into one fixation and fixations with durations shorter than 60 ms were excluded. The study extracted four widely used eye movement measures [16,44]: pupil diameter, fixation distance (i.e., distance between adjacent fixation points), blink count (number of blinks per second), and saccade count (number of saccades per second).

For preprocessing the raw ECG data, we removed noise and outliers. This involved wavelet denoising, bandpass filtering (0.01–200 Hz) to eliminate 50 Hz power line interference, setting a maximum heart rate threshold of 120 bpm, establishing an R-wave amplitude threshold of 70%, and defining a 20% threshold for premature beat detection. Ectopic intervals were corrected using the mean method. A total of four time-domain measures (average heart rate per minute (AVHR), inter-beat interval (IBI), standard deviation of heartbeat interval (SDNN), the square root of the mean of the sum of the squares of difference between successive R intervals (RMSSD)) and three frequency-domain measures (percentage of successive NN intervals that differ by more than 20 and 50 ms (pNN20, and pNN50), the rate of average power of low frequency to high frequency (LF/HF)) that were widely used for MWL assessment [15,17] were subsequently extracted.

As for the EDA signal, it underwent low-pass filtering at 0.02 Hz, wavelet denoising, and high-pass filtering at 0.2 Hz to preprocess the raw signal. This resulted in the extraction of two measures: skin conductance level (SCL), and skin conductance response (SCR).

#### 2.4.3. Driving Performance

Driving behavior data were recorded by the driving simulation system, which captured four parameters at a sampling rate of 20 Hz, including vehicle velocity, standard deviation of accelerator pedal input (SDoAPI), absolute value of rotation angles of steering wheel input (RAoSWI), and the absolute value of lateral movement of the vehicle from the central line of the lane (lateral position). 

In total, we obtained 23 objective measures to examine their association with MWL, including 4 EEG measures, 4 EOG measures, 7 ECG measures, 2 EDA measures, and 4 driving performance measures. The detailed description of the measures is shown in Table 1.

### 2.5. Data Analysis

Repeated measures ANOVA was used to examine the effects of task difficulty (low, medium, and high) on measures from subjective ratings, physiological measures, and behavioral performance. For measures with a significance level (*p* < 0.05), post hoc Tukey’s tests were conducted for pairwise comparisons. Pearson correlation analysis was performed to assess the interrelationships among the measures examined in this study. Pearson’s correlation analysis was employed to assess the strength of relationships between varied measures. A significant correlation coefficient close to 1 means a strong positive correlation between two measures, indicating that they exhibited similar trends in MWL assessment. In contrast, a significant correlation coefficient approaching −1 signifies a strong negative correlation between two measures, indicating that they exhibited opposite trends in MWL assessment. Conversely, a correlation coefficient near 0 suggests a lack of discernible linear correlation between the two measures. The significance level was set at *p* < 0.05. We completed the statistical analysis with SPSS 25.

## 3. Results

### 3.1. Subjective Ratings on MWL 

**Figure 2:**
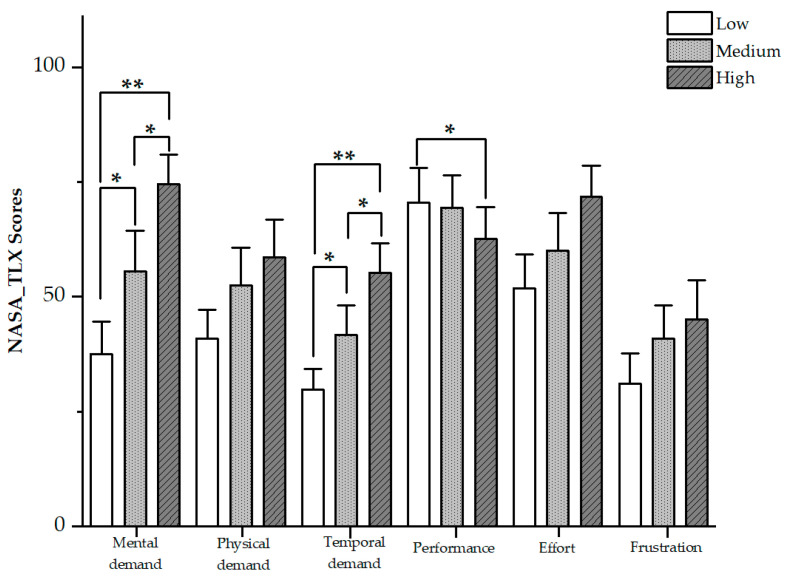
Comparisons of sub-dimensions of NASA-TLX among three tasks with different difficulty levels. Error bars represent standard errors (* *p* < 0.05, ** *p* < 0.01).

The overall MWL assessed by NASA-TLX was 32.36 (SD = 13.00), 41.95 (SD = 16.75), and 52.22 (SD = 13.29) for low, medium, and high difficulty tasks, respectively. There was a main effect of task difficulty on overall MWL (F(2,46) = 23.690, *p* < 0.001), indicating that the manipulation of task difficulty levels successfully induced different levels of MWL. Also, post hoc Tukey’s tests showed that there were significant differences between each pair of the three different tasks (*p*’s < 0.05). Specifically, statistical analysis revealed significant differences in the sub-dimensions of mental demand (F(2,46) = 26.806, *p* < 0.001) and temporal demand (F(2,46) = 21.319, *p* < 0.001) for each pair of the three different tasks as well. Performance was found to decrease as task difficulty increased (F(2,46) = 18.519, *p* < 0.05), while the decrease was shown to be significant only between low and high task difficulty levels (*p* < 0.05). Figure 2 presents the six sub-dimensions of NASA-TLX on MWL. For three other sub-dimensions (i.e., physical demand, effort, and frustration), they increased with task difficulty, although the increase did not show significant differences among the three different tasks.

### 3.2. EEG Measures

**Figure 3:**
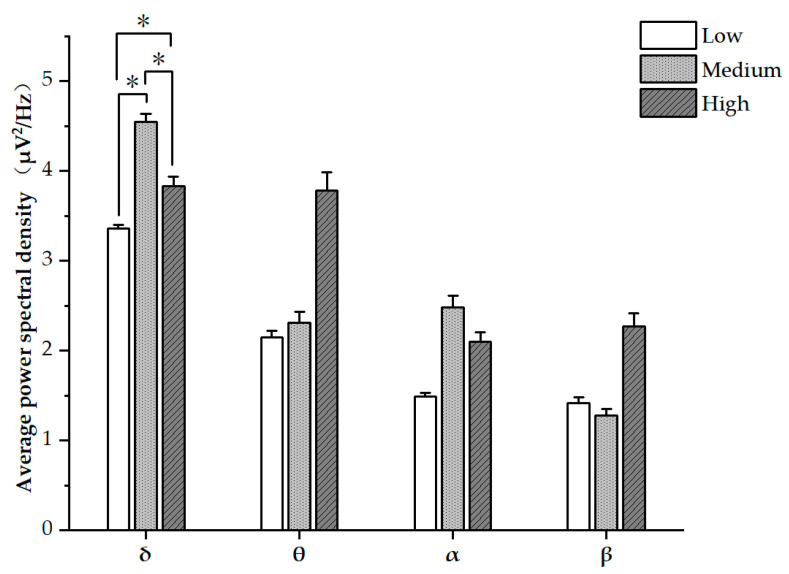
Comparisons of the four EEG measures among three tasks with different difficulty levels. Error bars represent standard errors (* *p* < 0.05).

There were significant differences in δ band PSD across low, medium, and high task difficulties (F(2,46) = 5.103, *p* = 0.010) (Figure 3, Table 2). Subsequent Tukey tests showed significant differences in δ band PSD across low, medium, and high task difficulties (*p*’s < 0.05). PSDs were found to be the highest in high task difficulty for θ and β, and highest in medium task difficulty for α. However, there were no significant differences observed in the PSDs of θ, α, and β bands. 

### 3.3. EOG Measures

Among the four EOG measures, only fixation distance demonstrated statistically significant differences across various task conditions (F(2,46) = 5.425, *p* = 0.015) (Table 2). As the MWL increased, the fixation distance decreased. Subsequent post hoc Tukey tests further revealed significant differences between the low and high difficulty levels (*p* < 0.05).

### 3.4. ECG Measures

As MWL increased, AVHR, SDNN, and RMSSD demonstrated an upward trend, while IBI, pNN20, and pNN50 showed a downward trend (Table 2). LF/HF did not display any significant pattern of change across different task levels. In particular, there was a significant main effect of task difficulty on AVHR (F(2,46) = 3.77, *p* = 0.030), IBI (F(2,46) = 2.11, *p* = 0.133), RMSSD (F(2,46) = 8.86, *p* = 0.002), and pNN50 (F(2,46) = 6.20, *p* = 0.004). Post hoc Tukey tests showed that, while AVHR IBI and pNN50 did not exhibit significant differences between medium and high difficulty levels, they showed significant differences between low and medium difficulty levels. RMSSD displayed significant differences between any two of the three task difficulty levels. No significant differences were found in SDNN, pNN20, and LF/HF among different task levels.

### 3.5. EDA Measures 

The values of SCL and SCR in medium-difficulty tasks were observed to be higher than those in low- and high-difficulty tasks (Table 2). The main effect of task difficulty on SCL (F(2,46) = 4.272, *p* = 0.020) and SCR (F(2,46) = 3.71, *p* = 0.032) was found to be significant. Post hoc Tukey tests revealed that significant differences existed in SCL between different difficulty levels of tasks. However, no significant difference was observed in SCR between the low and high difficulty levels.

### 3.6. Behavioral Performance

For secondary task performance, there was a significant decrease in accuracy (t = 4.390, *p* < 0.001) and a significant increase in response time (t = −6.712, *p* < 0.001), as the secondary task changed from a 1-back task to a 2-back task. This also indicated the successful manipulation of the task difficulty levels. For main task performance, there were significant differences among the four driving behavioral measures across different difficulty levels: velocity (F(2,46) = 0.500, *p* = 0.011), SDoAPI (F(2,46) = 10.151, *p* < 0.001), RAoSWI (F(2,46) = 5.865, *p* = 0.014), and lateral position (F(2,46) = 7.730, *p* < 0.001). Generally, it showed that the four measures decreased as task difficulty increased.

### 3.7. Correlations between Multimodal Measures

Pearson correlation analysis (Table 3) showed positive correlations among the four brain waves (δ, θ, α, β). Notably, θ wave exhibited the strongest correlation with α wave, with a correlation coefficient of 0.908. Conversely, the weakest correlation was observed between the β and δ waves, with a coefficient of only 0.295. Furthermore, PSD of δ waves showed notable negative associations with both pupil diameter and AVHR. Similarly, PSD of θ waves displayed significant negative correlations with AVHR and IBI. Conversely, α waves exhibited significant negative correlations with AVHR, while it demonstrated significant positive correlations with RMSSD, pNN20, pNN50, and SCR. Likewise, β waves exhibited comparable significant positive correlations with RMSSD, pNN20, pNN50, and SCR.

Regarding the EOG signals, pupil diameter exhibited noteworthy positive associations with fixation distance and SCL. Additionally, blink count displayed significant positive correlations with pNN20 and RAoSWI. Furthermore, saccade count demonstrated significant positive correlations with SCL. Concerning the ECG signals, AVHR showcased significant negative correlations with RMSSD, pNN20, pNN50, LF/HF ratio, SCL, SCR, and RAoWI. Moreover, IBI exhibited significant negative correlations with RMSSD, pNN20, and pNN50, while RMSSD demonstrated significant positive correlations with pNN20, pNN50, and LF/HF. The LF/HF ratio exhibited significant positive correlations with SCR. In terms of EDA signals, SCL exhibited a highly positive correlation with SCR, with a coefficient of 0.813, whereas SCR showed a significant positive correlation with RAoSWI. Among driving behavioral performance measures, SDoAPI demonstrated a significant positive correlation with velocity.

## 4. Discussion

Despite the extensive literature on the assessment of MWL, there are still knowledge gaps remaining in the utilization of different measurements to assess MWL in driving scenarios. Additionally, the effectiveness of multimodal measures was less investigated in similar contexts, making direct comparisons among them challenging. To address this issue, we proposed a multimodal approach for assessing MWL in a simulated auditory-based dual-task driving scenario. In particular, we aimed to explore whether a comprehensive set of measures, including subjective, physiological, and behavioral measures, can be utilized to assess changes in MWL. The subjective ratings validated that our manipulation of dual-task driving scenarios successfully induced different levels of MWL. Changes in task difficulty were associated with changes in many of the multimodal measures of MWL, which appears sensitive in reflecting MWL. The subsequent sections present a detailed discussion on the effectiveness of measures from different modalities in reflecting drivers’ MWL.

### 4.1. Subjective Ratings

The results demonstrate significant differences in subjective ratings of MWL among tasks with varying difficulty levels. This finding, consistent with previous studies that have used NASA-TLX to investigate subjective MWL [15,31], confirms that our manipulation of varied dual-task driving scenarios successfully induced varying levels of MWL.

However, our study also revealed some differences in outcomes compared to previous research. In particular, the sub-dimensions of mental demand, temporal demand, and performance contribute most to the significant differences in three levels of MWL. Our study showed a significant difference in the sub-dimension of performance only between low and high difficulty tasks, while Ding et al. [15] found that the performance dimension showed significant differences across low, medium, and high difficulty tasks. A possible explanation for this difference lies in the different task scenarios used in the two studies. Ding et al. induced task difficulty through mental arithmetic tasks, while our study involved the simultaneous processing of visual and low-difficulty auditory tasks. According to Wicken’s theory of multiple resources [12], visual and low-difficulty auditory tasks result in a limited degree of competitive allocation of mental resources. Our results suggest that the subjective rating of performance did not significantly change when drivers simultaneously processed the driving task and low-difficulty secondary tasks. Only when drivers needed to process higher-difficulty secondary task did their subjective performance rating decrease noticeably. These findings suggest that drivers believed that handling some simple voice information while driving would not contribute to their overall MWL.

Overall, subjective ratings of MWL in this experiment suggest that drivers do not perceive handling simple voice information while driving as contributing significantly to their overall MWL. This finding can inform the development of smart driving systems to minimize the complexity of voice information to alleviate the potential for driver distraction and enhance safety on the roads.

### 4.2. Physiological Measures

#### 4.2.1. EEG Measures

EEG evaluation has been established as an effective method for assessing MWL [45]. Numerous studies have shown that EEG rhythmicity is sensitive to changes in workload levels, for instance, Paxion et al. discovered that α waves are highly responsive to MWL, exhibiting a decrease in PSD as workload increases. Conversely, θ, β, and δ waves have demonstrated a positive correlation with workload intensity [18]. In our study, we aimed to further investigate the relationship between MWL and EEG patterns. We found that as MWL increased from low to medium, there were more pronounced changes in δ and α waves, while θ and β waves did not exhibit significant variations. As MWL escalated from medium to high, the PSD of θ and β waves increased, indicating a positive correlation, whereas δ and α waves decreased, displaying a negative correlation. These findings deviate somewhat from those of Hussain et al. [8], highlighting the limitations of relying solely on a single EEG indicator to assess MWL. It may be that different experimental scenarios may yield different results. 

Among the three levels of task difficulty, only δ waves exhibited significant differences in PSD changes among the various frequency brain waves. The patterns of change for three other brain waves varied significantly in our study. However, Borghini et al. [30] found that a decrease in α wave PSD was accompanied by an increase in θ wave PSD, as MWL increased. This aligns with the observed changes in α and θ waves when task difficulty changed from the medium level to the high level in our study. This also explains why some studies utilize multiple ratios of brain waves to evaluate MWL, such as the ratio of α to θ waves [46] and the ratio of θ to β waves [47].

#### 4.2.2. EOG Measures

Eye movement metrics provide insight into the activity of eyes, and can serve as measures of MWL in complex scenarios. Parameters such as pupil diameter, blink count, saccade count, and fixation distance can be analyzed to assess MWL [48]. However, the findings of this study did not reveal clear patterns in pupil diameter and saccade count across different levels of MWL. Nevertheless, blink rate was found to increase significantly with task difficulty, while fixation distance showed a significant decrease. 

Previous studies have established that eye activity can reflect MWL, but its manifestation is largely influenced by visual demands [49]. In our study, the secondary task involved limited visual demands, primarily relying on auditory-based presentation. This may explain why the observed variations in eye movement metrics were not pronounced. Consequently, the reliable eye movement metrics previously identified, such as pupil diameter [50], eye saccades [44], and blink rate [51], did not exhibit significant differences in this particular experiment.

These findings highlight the importance of considering task characteristics, particularly visual demands, when assessing MWL using eye movement metrics. Future research could explore the use of other measures or adaptations of eye movement metrics that are more suitable for scenarios with limited visual demands.

#### 4.2.3. ECG Measures

ECG measures are widely studied and are easily accessible measures. Portable devices, such as smart wristbands and watches, can capture cardiac indices like heart rate and heartbeat intervals. In this experiment, four temporal indices (AVHR, IBI, SDNN, RMSSD) and three frequency domain indices (pNN20, pNN50, LF/HF) were obtained from the cardiac signals. 

To provide a clear comparison between our findings and previous outcomes, we discussed each measure individually in relation to previous studies. For AVHR, De et al. reported an increase in AVHR with increased MWL in a simulated flight experiment [23]. However, in our study, AVHR only increased under low- and medium-difficulty levels, with no significant difference observed between medium- and high-difficulty levels. These findings differ from those of De et al. [23]. IBI is another commonly reported cardiac index in MWL assessment. A review by Tao et al. found significant differences in IBI among varying levels of MWL in 13 out of 19 previous studies [17]. In our study, we did observe a decrease in IBI with increased task difficulty, but this decrease did not reach statistical significance. For SDNN, although it changes with increased task difficulty, it did not show significant differences across different levels of MWL. Similar variations in SDNN were also observed by Hsu et al. [47]. For RMSSD, it was found to be the most sensitive ECG measure. Consistent with previous research [52], we found that it increased with higher MWL levels. Moreover, in our study, pNN50 decreases with increasing task difficulty. In particular, a decreasing trend was observed between low and medium MWL levels, similar to the findings of Wilson et al. [53]. This suggests that pNN50 may be more likely to be sensitive in low to medium MWL levels, and it may suffer from a ceiling effect in high MWL-level assessment. Finally, the ratio of LF/HF did not show significant differences at different levels of MWL, similar to the findings of Ding et al. [15], indicating that LF/HF may not be sensitive to MWL in both their and our study contexts.

In summary, our study reveals differences in AVHR and IBI compared to previous research, while SDNN, RMSSD, pNN50, and LF/HF show either similar or contrasting results. These findings also highlight the potential limitations and sensitivities of these ECG measures under varying task difficulties. The discrepancies can be attributed to various factors, including differences in experimental conditions, participant characteristics, and the specific measures employed. Therefore, it is crucial to conduct a comprehensive assessment of drivers’ MWL using multimodal measures during typical driving scenarios in order to gain a better understanding of the relationship between ECG indicators and MWL.

#### 4.2.4. EDA Measures

Previous studies have increasingly utilized EDA signals as a means of assessing MWL [17,19]. For example, the review by Charles et al. showed that 7 of 58 studies that used physiological measures on MWL assessment have employed EDA signals to gauge MWL [19]. This growing trend may be attributed to the fact that mental overload leads to an escalation in metabolic demand, resulting in stress and perspiration, thus causing variations in skin conductance signals.

Consistently, we found that EDA measures, such as SCL and SCR, exhibited significant main effects on MWL. In particular, these two measures increased as MWL increased from low to medium levels but showed a decreasing trend as MWL increased from medium to high levels. This result is consistent with findings of Mehler et al. [28]. They reported that an increase in skin conductance was observed with the augmentation of stimulus in the second task (to increase the task difficulty), but no change occurred when the task difficulty was further increased. This may indicate a lack of sensitivity for skin conductance signals in assessing higher MWL levels. This speculation might have been confirmed by Charles et al.’s review [19], which also highlighted the limitations of using skin conductance signals to assess higher MWL levels.

### 4.3. Behavioral Performance

Our study revealed significant differences in both the number of correct responses and reaction times between the 1-back and 2-back tasks. Furthermore, the results of the NASA-TLX questionnaire also indicated notable differences in subjective mental workload (MWL) levels across the various task difficulty levels, which validates our subtask design for inducing diverse levels of difficulty.

In addition, our investigation into the three different task levels demonstrated significant variations in driving performance among drivers. This was evidenced by the four measures we selected, including velocity, SDoAPI, RAoSWI, and lateral position. Specifically, as task difficulty increased, all four measures exhibited decreasing trends. These findings underscore that driving performance is highly sensitive to drivers’ MWL.

Moreover, our results suggest that as MWL increased, drivers became more cautious in their driving behaviors to counterbalance the negative impacts caused by MWL. This implies that drivers may be able to adapt their driving strategies to mitigate the effects of MWL on their driving performance. Overall, our study contributes to a better understanding of the relationship between MWL and driving performance and highlights the need for appropriate countermeasures to enhance driving safety.

### 4.4. Correlations among Multimodal Measures

The correlation analysis revealed significant associations among specific measures across different modalities, consistent with the findings reported in the review articles by Charles [21] and Huang [54]. These results suggested the widespread impact of MWL on various physiological aspects of the human body, indicating their varying degrees of sensitivity in reflecting MWL. Notably, a remarkably strong correlation was observed among the EEG measures, while pupil diameter in eye movement signals exhibited robust correlations with other eye movement characteristics. In contrast, heart rate in ECG signals displayed pronounced negative correlations with other ECG measures. Furthermore, a substantial positive correlation was noted between skin conductance level and skin conductance response in the EDA signals. Some measures within the driving behavioral performance also demonstrated correlations.

Overall, the correlations among measures within the same modality were more prominent than those from different modalities. However, no discernible pattern emerged regarding the correlations among measures from different modalities. These findings partially align with the ANOVA results, indicating that different modal measures exhibit varying sensitivity to MWL. This appears to suggest the presence of collinearity or redundant measurements when evaluating MWL solely using measures from the same modality or a single modality. It also emphasized the need for future research to adopt multimodal measurement approaches for a more comprehensive assessment of MWL.

### 4.5. Implications

The findings from this study have significant implications for the evaluation and prediction of MWL in the driving industry. First, our study established a multimodal approach for drivers’ MWL assessment through a typical auditory-based dual-task driving scenario. Data on a comprehensive set of measures, including subjective evaluations, physiological measures, and behavioral performance, were synchronously recorded, which allows for direct comparison among the multimodal measures about the sensitivity to drivers’ MWL. 

Second, various physiological measures, including EEG, EOG, ECG, and EDA, showed diverse responses to task difficulty, suggesting that each type of measure may offer distinct insights into MWL assessment. This highlights the necessity to take into account multiple types of physiological measures to accurately assess MWL in specific contexts. Furthermore, significant differences in specific measures were identified across three levels of task difficulty, emphasizing the limitations of relying solely on individual physiological measures to comprehensively reflect MWL characteristics. Therefore, a comprehensive approach considering multimodal measures, such as the δ wave of the EEG, fixation distance of the EOG, RMSSD, and pNN50 of the ECG, and SCL of the EDA, is crucial for an accurate assessment of MWL. Future research should prioritize the rational and effective utilization of these measures to establish a mechanism for multimodal MWL assessment.

Finally, integrating these findings into future research and practical applications can aid in the development of real-time tools for continuous monitoring and predicting MWL in future development of smart driving systems, thereby preventing accidents caused by mental overload, and ultimately improving road safety. Specifically, a comprehensive assessment using multiple physiological measures can assist IVIS in perceiving drivers’ MWL more accurately, thus enabling the realization of more intelligent and personalized driving assistance systems. For example, when a driving system detects excessively high MWL in a driver, it can implement corresponding intelligent assistance via IVIS, based on specific changes in physiological measures. Such assistance may involve adjusting the vehicle’s interior environment, providing voice prompts, or automating certain operations to help alleviate the driver’s MWL. Consequently, these findings have significant implications for the development of intelligent-assisted driving systems. Future research can further explore the integration of multiple physiological measures with IVIs to achieve precise prediction and intelligent assistance for drivers’ mental workload.

### 4.6. Limitations

There are limitations to this study. First, due to safety concerns, a driving simulation environment was utilized, limiting the replication of real driving processes. Unlike what occurs in highly controlled laboratory environments, real-world environments are more dynamic and complex, and drivers’ physiological reactions could be different from those in a laboratory driving environment [55,56]. Therefore, future studies should consider utilizing a real driving environment while ensuring driving safety. Second, due to the inconvenience of females wearing the wearable sensors and devices, as well as the recruitment of participants from campus, our participants were predominantly young males aged 20–30, with little variation in their driving experience. However, driving experience might be an important factor in drivers’ responses to different levels of mental workload [55]. Future studies could extend our study to include a more diverse population (e.g., including individuals with both genders and with diverse driving experience) in order to consider the potential impact of gender and driving experience on the experimental results. Finally, the measures used in this experiment represent average values over a certain period, without considering temporal changes in MWL [32]. Thus, future research could explore the temporal characteristics of MWL and examine how MWL would change over time. 

## 5. Conclusions

This study was conducted to provide a comprehensive assessment of drivers’ MWL by multimodal measures through a typical auditory-based dual-task driving scenario. The findings demonstrated that the NASA-TLX effectively measures the intensity of MWL in varied dual-task driving scenarios, albeit it typically served as a validation method due to its post-test nature. Multimodal physiological signals, including EEG, EOG, ECG, and EDA measures, were analyzed across varied task difficulty levels, and they exhibited diverse trends in response to task difficulty. Several measures that were effective in drivers’ MWL assessment were identified, includingδ wave in EEG signals, fixation distance in EOG signals, RMSSD and pNN50 in ECG signals, and SCL in EDA signals. The findings from this study can help establish a multimodal approach for drivers’ MWL assessment and can provide valuable insights for the assessment of MWL in the driving industry and the development of smart driving systems for the accurate recognition of drivers’ mental states.

## Figures and Tables

**Table 1:** Description of indexes measured in the experiment.

Modalities	Measures	Unit	Description
EEG	δ	μV^2^/Hz	Power spectral density (PSD) of delta power (1–4 Hz)
	θ	μV^2^/Hz	PSD of theta power (4–8 Hz)
	α	μV^2^/Hz	PSD of alpha power (8–14 Hz)
	β	μV^2^/Hz	PSD of beta power (14–30 Hz)
EOG	Pupil diameter	mm	Diameter of the pupils
	Fixation distance	px	Distance between adjacent fixation points
	Blink count	N/s	Number of blinks per second
	Saccade count	N/s	Number of Saccades per second
ECG	AVHR	bpm	Average heart rate per minute
	IBI	ms	Inter-Beat Interval
	SDNN	ms	Standard deviation of heart beat interval
	RMSSD	ms	The square root of the mean of the sum of the squares of difference between successive R intervals
	pNN20	%	Percentage of successive NN intervals that differ by more than 20 ms
	pNN50	%	Percentage of successive NN intervals that differ by more than 20 ms
	LF	ms^2^	Average power of low frequency (0.04–0.15 Hz)
	HF	ms^2^	Average power of high frequency (0.15–0.4 Hz)
	LF/HF	-	The rate of LF to HF
EDA	Skin conductance level (SCL)	μS	The average conductivity level on skin surface during a set time interval
	Skin conductance response (SCR)	μS	The phasic difference in skin’s moisture level before and after presentation of a stimulus
Driving performance	Velocity	km/h	Average velocity
	SDoAPI	-	Standard deviation of accelerator pedal input
	RAoSWI	(°)	Absolute value of rotation angles of steering wheel input
	Lateral position	m	Absolute value of lateral movement of the vehicle from the central line of the lane

**Table 2:** Statistical results of physiological measures and driving performance (mean ± standard deviation).

Modalities	Measures	Low	Medium	High	F	*p*
EEG	δ (μV^2^/Hz)	3.36 ± 0.19 ^a^	4.55 ± 0.43 ^b^	3.83 ± 0.53 ^c^	5.103	**0.010**
	θ (μV^2^/Hz)	2.15 ± 0.34	2.31 ± 0.61	3.78 ± 1.00	3.277	0.069
	α (μV^2^/Hz)	1.49 ± 0.21	2.48 ± 0.64	2.10 ± 0.51	2.259	0.138
	β (μV^2^/Hz)	1.42 ± 0.31	1.28 ± 0.34	2.27 ± 0.72	2.060	0.162
EOG	Pupil diameter (mm)	3.53 ± 0.57	3.36 ± 0.79	3.61 ± 0.64	1.934	0.176
	Fixation distance (px)	220.24 ± 69.31 ^a^	204.57 ± 71.96 ^ab^	175.98 ± 67.2 ^b^	5.425	**0.015**
	Blink count (N/s)	0.46 ± 0.28	0.80 ± 0.28	0.52 ± 0.28	1.353	0.269
	Saccade count (N/s)	4.05 ± 2.76	3.46 ± 2.60	3.50 ± 2.18	1.361	0.265
ECG	AVHR (bpm)	79.50 ± 13.78 ^a^	89.07 ± 13.00 ^b^	89.18 ± 13.59 ^b^	3.77	**0.030**
	IBI (ms)	706.22 ± 132.27 ^a^	676.37 ± 86.29 ^b^	636.18 ± 98.98 ^b^	2.11	**0.133**
	SDNN (ms)	282.70 ± 91.40	314.24 ± 78.10	419.42 ± 151.93	0.20	0.608
	RMSSD (ms)	40.13 ± 13.83 ^a^	54.58 ± 30.48 ^b^	76.61 ± 43.72 ^c^	8.86	**0.002**
	pNN20 (%)	0.62 ± 0.20	0.54 ± 0.17	0.53 ± 0.14	2.446	0.098
	pNN50 (%)	0.49 ± 0.27 ^a^	0.32 ± 0.12 ^b^	0.33 ± 0.18 ^b^	6.20	**0.004**
	LF/HF	4.88 ± 6.11	4.53 ± 2.86	5.12 ± 6.76	0.076	0.927
EDA	SCL (μS)	4.49 ± 1.55 ^a^	6.57 ± 4.79 ^b^	5.39 ± 2.54 ^c^	4.272	**0.020**
	SCR (μS)	0.14 ± 0.08 ^a^	0.20 ± 0.16 ^b^	0.14 ± 0.07 ^a^	3.710	**0.032**
Driving performance	Velocity (km/h)	80.94 ± 3.38 ^a^	78.70 ± 3.46 ^b^	79.09 ± 3.78 ^b^	0.500	**0.011**
	SDoAPI	0.23 ± 0.10 ^a^	0.18 ± 0.07 ^b^	0.17 ± 0.07 ^b^	10.151	**<0.001**
	RAoSWI (°)	1.61 ± 0.32 ^a^	1.57 ± 0.23 ^a^	1.45 ± 0.23 ^b^	5.865	**0.014**
	Lateral position (m)	0.49 ± 0.12 ^a^	0.45 ± 0.13 ^ab^	0.42 ± 0.13 ^b^	7.730	**<0.001**

Note: Values labelled with different superscript letters for groups in the same measures indicate a significant difference (*p* < 0.05) as revealed by the Tukey test. Bold/underline indicates a statistically significant difference (*p* < 0.05).

**Table 3:** Correlation analysis results among multimodal measures.

Measures	δ	θ	α	β	Pupil Diameter	Fixation Distance	Blink	Saccade	AVHR	IBI	RMSSD	pNN20	pNN50	LF/HF	SCL	SCR	Velocity	RAoSWI	SDoAPI	Lateral Position
δ	1																			
θ	0.897 *	1																		
α	0.720 *	0.908 *	1																	
β	0.295 *	0.555 *	0.786 *	1																
Pupil diameter	−0.244 *	−0.197	−0.160	0.022	1															
Fixation distance	−0.134	−0.148	−0.136	−0.070	0.387 *	1														
Blink	−0.025	−0.030	−0.034	−0.027	−0.101	−0.001	1													
Saccade	0.107	0.087	0.052	0.030	0.157	−0.193	−0.046	1												
AVHR	−0.266 *	−0.318 *	−0.352 *	−0.231	0.051	0.015	−0.180	0.050	1											
IBI	−0.192	−0.296 *	−0.215	−0.159	−0.114	−0.221	−0.180	−0.206	−0.421	1										
RMSSD	0.135	0.208	0.267 *	0.245 *	−0.062	−0.149	0.144	0.005	−0.835 *	−0.244	1									
pNN20	0.044	0.148	0.246 *	0.264 *	0.019	−0.039	**0.252**	0.023	−0.522 *	−0.276 *	0.502 *	1								
pNN50	0.058	0.152	0.257 *	0.270 *	0.190	0.054	0.222	0.062	−0.564 *	−0.359 *	0.376 *	0.889 *	1							
LF/HF	0.032	0.062	0.117	0.164	−0.033	−0.199	0.164	0.188	−0.528 *	−0.528	0.748 *	0.211	0.149	1						
SCL	−0.07	−0.011	0.049	0.049	0.145 *	−0.004	−0.075	0.179 *	−0.166 *	0.007	0.145	0.045	0.17	0.076	1					
SCR	0.033	0.075	0.131 *	0.128 *	0.051	0.007	−0.057	0.027	−0.176 *	−0.059	0.070	0.003	−0.121	0.110 *	0.813 *	1				
Velocity	0.106	0.060	−0.059	−0.097	0.135	0.115	0.008	−0.001	0.122	−0.004	−0.106	−0.055	−0.063	−0.114	−0.142	0.051	1			
RAoSWI	0.136	0.124	0.151	0.060	−0.059	−0.169	0.232 *	−0.020	−0.321 *	0.107	0.316 *	0.213	0.150	0.223	0.165	0.122 *	−0.011	1		
SDoAPI	0.114	0.072	−0.014	−0.144	−0.177	−0.053	0.198	−0.001	−0.145	0.033	0.040	0.028	−0.036	−0.057	−0.018	0.079	0.277 *	0.358 *	1	
Lateral position	−0.145	−0.139	−0.145	−0.108	0.152	−0.087	0.046	0.009	0.048	−0.119	−0.002	0.016	−0.036	0.007	−0.106	0.003	−0.011	−0.031	−0.036	1

* *p* < 0.05. Bold/underline indicates a statistically significant difference (*p* < 0.05).

## Data Availability

The data presented in this study are available on request from the corresponding author. The data are not publicly available due to privacy issue.
